# Experimental Researches Applied to Physiology and Pathology

**Published:** 1852-11

**Authors:** E. Brown-Séquard

**Affiliations:** Paris


### *Experimental Researches applied to Physiology and Pathology.*

By E. Brown-Séquard, M. D., of Paris.

(Continued.)

### XIX.—ON THE MODE OF ACTION OF SOME OF THE MOST ACTIVE POISONS UPON THE NERVOUS SYSTEM.

Some of the results of my experiments on this subject have already been published in the inaugural dissertation of my learned friend and pupil, Dr. F. W. Bonnefin, with whom I performed most of these experiments.*

I intend to give here only the results of my researches on the poisons which produce convulsions. For the sake of brevity I have called them *convulsing* poisons. The most important among them are: strychnine, brucine, picrotoxine, morphine, digitaline, cyanhydric acid, nicotine, cyanide of mercury, sulphide of carbon, chloride of barium, and oxalic acid.

The principal questions which I have endeavored to solve are the following:

1. What is the part of the animal frame upon which these poisons act in producing convulsions?

2. By what mode of action do they produce convulsions?

The parts of the body on which they could act are:—

*a.* The nervous centres.

*b.* The motor or centrifugal nerves.

*c.* The sensitive or centripetal nerves.

*d.* The muscles.

Of course, the convulsing poisons could act upon these four parts together, or upon two or three of them.

Now, as to the mode of action, these poisons could produce convulsions *directly* or *indirectly*.

In other words these poisons might act:—1. As *excitants* either of the muscles or of the nervous system, and this is what I call a direct action. 2. As causes of increase of the vital powers of the muscles or of the nervous system, so that even a slight excitation of these parts is able to produce convulsions, and this is what I call an indirect action.

* Rech. Expérim. sur l'action convulsivante des principaux Poisons, in 4 Paris, 1851.

The convulsing poisons do not appear to excite the muscles or any part of the nervous system, and consequently they do not act directly. They act merely in increasing the vital powers of the nervous system, so that a slight excitation of the skin or another part where there exist nerves of sensation is sufficient to produce convulsions. Therefore these poisons act indirectly in the production of convulsions. They do not produce convulsions, they merely act upon the nervous system, so as to render it capable of producing convulsions when it receives an excitation. They do not excite the nervous system, and their mode of action is altogether different from that of the mechanical, physical, and chemical excitations which directly produce convulsions, when they are applied to the medulla oblongata, or to the spinal cord.

This mode of action was already known as regards strychnine. Long ago Mr. Magendie* found that animals poisoned by *nux vomica*, frequently remain without convulsions as long as they are left without excitation, but that fits are immediately produced when they are touched. Since that time Stannius,† Van Deen,‡ Pickford,|| J. W. Arnold,§ Meyer, of Zurich,¶ Marshall Hall,** and A. Barnard,†† have observed many facts proving that strychnine does not directly produce convulsions. The following experiment, which I performed with the assistance of Dr. Bonnefin, is still more demonstrative.

I introduce a large dose of strychnine into the stomach of a frog, after the removal of its brain and of its medulla oblongata. As the voluntary and the respiratory movements are then impossible, there is no spontaneous movement at all, and if the animal is left perfectly quiet there are no convulsions. But as soon as it is excited, even by the slightest touch, tetanus occurs.

In this case, it is evident that the convulsive fit is merely a re-

* Examen de l'action de quelques végétaux sur la Moelle épinière. Paris 1809, p. 7.

† Mueller's Archiv. 1837, p. 223.

‡ Traités et découvertes sur la Moelle épinière, 1841, p. 123,

|| In Rorer und Wrarderlich's Vierteljaheschrift. Bd. 2, 1843, p. 430.

§ Ueber die verrichtung der Werzeln der Rückenmarksnerven, 1844.

¶ Schmidt's Jahrbücher, 1847, No. 8.

** Comptes rendus de l'Acad. des Sciences, 1847, vol. 24, p. 1054.

†† Procès verbaux de la Société Philomatique, 1847, p. 71.

flex act. But why is there a tetanic contraction instead of the regular reflex movements? The reason is that the reflex faculty is considerably increased. This will be proved in a moment.

Before giving this demonstration, I must examine if there is not also an increase in the vital powers of the muscles and of the motor nerves. There was no reason to reject the supposition that the convulsing poisons were capable of increasing simultaneously the vital powers of the nervous centres, of the nerves, and of the muscles. Consequently, I was led to perform the following experiments, which prove:—1st, that these poisons do not increase the vital powers of the motor nerves and of muscles; 2d, that they do not act as direct excitants upon these organs.

On young cats, on birds, and on reptiles, I removed the whole portion of the spinal cord which supplies nerves to the posterior limbs. A few minutes after, I injected into the rectum a solution of a salt of strychnine. Convulsions occurred only in the anterior parts of the body, and when I excited either the motor nerves or the muscles of the posterior limbs, contractions were produced exactly as in animals not poisoned, but there was no appearance of convulsions.

When the poison was placed directly on muscles or on nerves, or when it was injected with blood in a limb separated from the body, there was no appearance either of an excitation, or of an increase in the excitability of the muscles, or of the motor nerves.

The experiments of Magendie, of Emmert,* and of Backer,† have demonstrated that after a transverse section of the spinal cord, between its two enlargements, convulsions may be produced in the palsied limbs, when the animal is poisoned with strychnine. All the physiologists who have performed this experiment have found it perfectly exact. Nevertheless, J. W. Arnold,‡ maintains that the action is much less considerable in the posterior limbs, in that case, than when the spinal cord is uninjured and united with the medulla oblongata, and he concludes that the poison

* Exper. de effectu venenorum veget. americ. in corpus animale, 1817.

† Commentatio ad questionem physiol., ab Acad. Rheno traject, anno 1828, propositam.

‡ Die lehre von der Reflex-function. Chap. ix. and x. 1842.

acts much more on the medulla oblongata than on the spinal cord.

Sometimes, as Arnold says, it occurs that the action of strychnine, in mammalia and in amphibia, is not so powerful in the palsied parts, after the section of the spinal cord, as in the non-palsied limbs. As to mammalia, the reason of this difference is the diminution in the quantity of blood received by the part of the spinal marrow separated from the encephalon. As to amphibia, it is easily seen that immediately after the section of their spinal cord, the reflex faculty in the part separated from the encephalon is very weak, and if, then, strychnine is given to the animal, it does not act very strongly; but if the poison is given two or three hours after the section of the spinal cord, then the reflex faculty is very powerful and the poison acts violently, and sometimes more than if the spinal cord was uninjured. In birds, which, as I have discovered, have constantly a powerful reflex faculty after the section of the spinal cord, strychnine acts very energetically.

Lately, Stannius and Cl. Bernard have supposed that strychnine, instead of acting on the spinal marrow, acted on the nerves of sensibility, and more particularly on their termination in the skin. They base this hypothesis on some experiments, of which only one is important. After the section of the spinal cord, at the brachial enlargement, upon a frog, the animal is poisoned with strychnine, and then convulsions occur nearly at each voluntary or respiratory movement. But if the sensitive roots of the spinal nerves are cut, the convulsions cease immediately.

Now, it is evident that this fact does not prove what Stannius and Bernard have supposed; because it may be explained as well by admitting that convulsions are produced only in consequence of an increase of the reflex faculty of the spinal cord, as by the hypothesis of Stannius.

Van Deen * relates an experiment which is in opposition to the theory of Stannius. If we take a frog prepared exactly as in the experiment of this physiologist, we see that tetanus occurs when the animal is thrown on the floor. In this case tetanus is produced, although the sensitive roots are cut and unable to act; therefore tetanus is a consequence of an increased

* Loco cit. p. 123.

vitality in the spinal cord itself. But this experiment is not decisive, because sometimes it occurs that such a mechanical excitation in frogs, which have not been poisoned, produces tetanus. Nevertheless I must say that the tetanus in this last case is never so violent as in poisoned frogs.

A better experiment is to excite slightly with a needle, the posterior columns of the spinal cord. Then, tetanus constantly occurs in poisoned frogs, although the sensitive roots are divided, and it very rarely occurs in frogs that are not poisoned.

I have already published* the following experiments, which are much more decisive against the hypothesis of Stannius:

A ligature is put around the aorta of a frog, near its termination in the abdomen, and consequently the posterior limbs cease to receive blood. Then the frog is poisoned with strychnine introduced into its mouth, and after a few minutes the convulsive phenomena take place.

In this experiment the nerves of the posterior limbs do not receive strychnine, whilst the spinal cord receives it; therefore convulsions are not produced in consequence of an action of strychnine on the sensitive nerves of the skin, as Stannius has supposed, but in consequence of its action on the spinal cord.

Now, if we poison a frog after having divided the spinal cord at the brachial enlargement, and after the section of all the small arteries giving blood to the spinal column, we see that convulsions do not take place in the posterior limbs, although the reflex faculty is not lost in consequence of the cessation of the circulation in the spinal cord, and that it remains for half an hour or a little more in summer, and about two hours in winter.

In this experiment, blood containing strychnine reaches the sensitive nerves of the posterior limbs, and not the spinal cord, and there are no convulsions; therefore it is not on the sensitive nerves that strychnine acts in order to produce convulsions.

These two experiments are evidently decisive. In the first, we see that when blood containing strychnine reaches the spinal cord, and not the cutaneous nerves, there are convulsions; and in the second, we see that when blood containing strychnine reaches the sensitive nerves and not the spinal cord, there

* Gaz. Med. de Paris, 1849, p. 745.

are no convulsions. We must consequently draw from these experiments these two conclusions:

1. Strychnine does not act upon the sensitive nerves.

2. Strychnine acts upon the spinal cord.

Now, from all the facts above related, two other conclusions are to be drawn:

1. Strychnine does not excite the nervous system; or, in other words, strychnine does not produce convulsions directly.

2. Strychnine increases the reflex faculty of the spinal cord, and so produces convulsions indirectly.

I do not intend to examine here whether strychnine kills in producing convulsions, or by another action. Nevertheless, I will say, that although convulsions are sufficient to kill in asphyxiating, death, in cases of poisoning by strychnine, may be also produced by another action of that poison. I have seen animals in which convulsions did not take place at all, and which have been killed by strychnine.

The other convulsing poisons that I have studied, appear to act as strychnine, as to the production of convulsions.

The same experiments which I have related as regards strychnine, have been performed with these poisons, and I have obtained the same results. Sometimes, nevertheless, I found some differences; and, for instance, it appears that the chloride of barium is a direct exciter of the muscular fibres, and cyanhydric and oxalic acids seem also to be slight but direct exciters of the spinal cord.

The action of the chloride of barium is very important, because that poison is an exciter of the muscular fibres, and not of the nerves. This fact proves that the muscular irritability may be put in action without the intervention of the nerves.

The increase of the reflex faculty, by the convulsing poisons, is a very important fact. How is that increase produced? We believe it takes place in consequence of an increase in the nutrition of the nervous centres. J. Mueller* is of opinion that there is no substance able to increase directly the vital properties of any organ. He says that nutrition alone is able to produce such an effect. I believe he is perfectly right, and I admit that the mode of action of the convulsing poisons, in the

* Manuel de Physiol., edited by Littre, 1851, t. i. p. 582.

production of convulsions, is merely to increase nutrition in the nervous centres. It is important for practitioners to know that mode of action. The usefulness of strychnine in many cases of palsy, may be explained very easily by that action. I have frequently seen, in the wards of the hospital *la Charité* at Paris, paralytics under the care of Dr. Rayer, taking strychnine. Every day the reflex faculty was increasing in them as long as they took that substance; and on the contrary, when the use of that medicament was stopped, the reflex faculty began immediately to diminish, and in some patients it disappeared. If strychnine was given anew, the reflex faculty was still increased. These facts have been recorded with great care by my learned friend, Mr. Chareot. I hope he will publish them.

From all the facts narrated in this paper, I believe I am entitled to draw the following conclusions:

1. The convulsing poisons, more particularly strychnine, brucine, picrotoxine, cyanhydric acid, nicotine, morphine, cyanide of mercury, sulphide of carbon, digitaline, oxalic acid, appear to produce convulsions, without acting either directly or indirectly on the muscles or on the motor or sensible nerves.
2. Generally these poisons do not appear to produce convulsions in acting directly on any part of the nervous centres.
3. These poisons, in producing convulsions, act only on the parts of the nervous system endowed with the reflex faculty.
4. The mode of action of these poisons consists in the increase of the nutrition of the nervous centres, by which excess of nutrition the reflex faculty becomes much increased.

### XX.—ON THE CROSSED TRANSMISSION OF IMPRESSIONS IN THE SPINAL CORD.

Numerous experiments which I have performed have proved to the numerous physicians and students, who have seen the most important of them, that the impressions made on one side of the body are transmitted to the sensorium by the opposite side of the spinal cord.

It is known that Galen* performed two experiments, which

*See: De locis affectis, lib. iii. cap. xiv; or De anatomicis admonstrationibus, lib. viii. sect. 6.

have been considered as demonstrating that there is no *crossed* action in the spinal cord.

One of these experiments of Galen consisted in the transversal section of a lateral half of the spinal marrow. After this operation the animal was paralyzed in all the parts situated behind the section, on the same side, so that the palsy was on the right side of the body when the right side of the spinal cord was divided, and *vice versa.*

The second experiment consisted in a longitudinal section on the middle line of the spinal cord so as to separate into two lateral halves the part of that nervous centre supplying nerves to the posterior limbs. After this operation the animal was able to walk.

Galen, in these two experiments did not examine the state of the sensibility. He speaks merely of the voluntary movements. Nevertheless his researches were considered in this century as completely proving that there is no crossing of action in the spinal cord, either for sensibility or for voluntary movement.

The following experiments will prove that there is a crossing of action for sensibility in that organ:

1st. If a lateral half (*i. e.* the posterior and the antero-lateral columns and the gray matter of one side of the spinal cord), is divided transversely at the level of the tenth costal vertebra, on a mammal, it is soon evident that the sensibility is much diminished in the posterior limb opposite to the side of the sections. On the contrary the sensibility instead of being lost appears much increased in the posterior limb on the side where the section has been made.

2d. If, instead of one transversal section of the spinal cord, two, three, four or many more are made on the same lateral half of that organ, the same results are obtained.

3d. If, instead of mere sections, a removal of a part of a lateral half of the spinal cord, is effected, the same results are still obtained. In performing this experiment a longitudinal section, one inch in length, from behind forward, is made in the median plane of the spinal marrow, and then two transversal sections on a lateral half are made at the extremities of the longitudinal section, so that a part of the cord is completely separated from that organ and afterwards removed.

4th. If instead of dividing entirely a lateral half of the spinal cord, a small part is left undivided towards the centre of that organ, the posterior limb on the same side becomes much more sensible, but the posterior limb on the opposite side remains very sensible and sometimes it appears more sensible than in the normal state.

5th. If in performing the section of a lateral half of the spinal cord the instrument goes a little too far and divides also a small portion of the other half, then the posterior limb on the side of the complete section is less sensible than in the normal state, and the posterior limb of the opposite side, loses completely its sensibility.

6th. If the section of a lateral half of the spinal cord is made at the level of the second or third cervical vertebra, it is found that the sensibility becomes very quickly much greater in the parts of the body on the same side as the section, and on the contrary the parts on the other side becomes evidently less sensible.

7th. If after a section of a lateral half of the spinal cord at the level of the eleventh costal vertebra, another section is performed on the other side of that organ, at the level of the sixth costal vertebra, so that the two lateral halves are divided, then sensibility in most of the cases is lost, on both sides. Sometimes it retains a very slight degree of sensibility, more particularly in the posterior limb on the side where the spinal cord has been divided at the level of the sixth costal vertebra.

8th. If two sections of lateral halves are made as in the preceding experiment, but at a greater distance, one from the other, on the right side for instance at the level of the twelfth costal vertebra, and on the left side in the cervical region, nearly the same results are obtained as to the posterior limbs, but the sensibility is increased in the right anterior limb and it remains very evidently, but much diminished, in the left anterior limb.

9th. If a longitudinal section is made on the part of the spinal cord giving nerves to the posterior extremity, so as to divide that part into two lateral halves, then it is found that sensibility is completely lost in the two posterior limbs, although voluntary movements take place in them.

10th. If a similar separation of two lateral halves of the spinal cord is made on the whole part supplying nerves to the anterior limbs, then it is found that sensibility is lost in both these limbs, and that it is only slightly diminished in the posterior limbs.

11th. If the same operation is done as in the preceding experiment, and afterwards if a transversal division is made on one of the lateral halves, in the place where it is separated from the other, then it is found that the posterior limb on the side of the transversal section remains sensible, and that the other posterior limb loses its sensibility.

These experiments prove very clearly that the sensitive nervous fibres are crossed in the spinal cord. The 9th, 10th, and 11th, demonstrate directly the crossing. In these experiments the crossed fibres are all cut, and sensibility is lost. This fact appears to prove that all the sensitive fibres cross each other; but it will be easily understood that on account of the loss of blood, and of the general diminution of sensibility produced by the excessive pain of the operation, if there are some fibres which remain without crossing, they are insufficient to give sensations.

As to the experiments consisting in transversal sections of a lateral half, they prove that sensibility is much diminished in the side of the body opposite to that of the section; consequently they prove also that there is a crossing of a great part of the sensitive fibres.

The fact that transmission of impressions made on one side of the body takes place, at least for a great part, in the opposite side of the spinal cord, is proved evidently by the eight first experiments, but much more by the 7th and the 8th experiments in which it is found that, after a section of a lateral half of the spinal cord, sensibility remains on the same side, and that it is nearly entirely lost after a second section of the other lateral half in another place.

If most of the nervous sensitive fibres are crossed in the spinal cord, then it is not exact to admit that the crossed paralysis of sensibility in cases of diseases of the brain, is explained by the crossing of fibres which exists in the pons varolii and in other parts of the encephalon. Many opinions have been proposed as regards the place where the sensitive nervous fibres make their crossing in the encephalon. According to some pathologists, this crossing takes place all along the medulla oblongata, the pons Varolii, tubercula quadrigemina and the crura cerebri. In all these organs there is truly a crossing of fibres, but we do not know what are these fibres. Ch. Bell believes that the crossing of the sensitive fibres takes place in the posterior surface of the medulla oblongata, in a great part of the length of the fourth ventricle. Longet supposes that this crossing exists at the anterior border of the pons Varolii, where the two *processi cerebelli ad testes* cross each other.

My experiments prove that if there are some fibres coming from the trunk and from the limbs which do not effect their crossing in the spinal cord itself, their number ought to be very small. Therefore the fibres which are found crossed in the encephalon are not sensitive fibres coming from the limbs and from the trunk, as all physiologists have supposed they were.

My experiments were made on many different species; guinea-pigs, dogs, cats, sheep, and rabbits. In all the same results were obtained.

To ascertain the degree of sensibility, I used various modes of excitation; mechanical, galvanic, physical, (*i. e.* warmth and cold,) and chemical. I constantly compared the degrees of sensibility in the parts of the body situated behind the injured portion of the spinal cord, with the anterior parts of the body, and particularly with the face. It is thus that I have been able to ascertain the existence of an increase or of a diminution in sensibility.

Sometimes I have given chloroform to animals having had a lateral half of the spinal cord divided in the cervical region. I have found that complete loss of sensibility appeared at first in the parts of the body opposite to the section of the spinal cord, the head and neck, and at last in the parts of the body behind the section of the cord, on the same side. This experiment, as well as many others, prove undoubtedly that there is an increase of sensibility in these last parts. I will try in another article to explain this hyperæsthesia.

I believe I am entitled to conclude from the facts above related:

1st. That most of the impressions made on one side of the body are transmitted to the sensorium by the opposite side of the spinal cord, so that the impressions on the left side of the body are transmitted by the right side of the spinal cord, and *vice versa.*

2d. That the assumed function of the crossing of fibres in the pons Varolii, and the neighboring parts, does not belong to these fibres, but to the fibres of the spinal cord, all along which they cross each other.

